# Magneto-Optical Nanostructures for Viral Sensing

**DOI:** 10.3390/nano10071271

**Published:** 2020-06-29

**Authors:** Sabine Szunerits, Tamazouzt Nait Saada, Dalila Meziane, Rabah Boukherroub

**Affiliations:** 1 Institut d’Electronique, de Microélectronique et de Nanotechnologie (IEMN-UMR CNRS 8520), University Lille, CNRS, Centrale Lille, University Polytechnique Hauts-de-France, UMR 8520-IEMN, F-59000 Lille, France; nait.azouzou@hotmail.fr (T.N.S.); rabah.boukherroub@univ-lille.fr (R.B.); 2 Laboratory of Applied Chemistry and Chemical Engineering (LCAGC), Université Mouloud Mammeri de Tizi-Ouzou, Tizi-Ouzou -15000, Algeria; d_meziane@yahoo.fr

**Keywords:** virus, sensing, plasmonic, magnetic nanoparticles

## Abstract

The eradication of viral infections is an ongoing challenge in the medical field, as currently evidenced with the newly emerged Coronavirus disease 2019 (COVID-19) associated with severe respiratory distress. As treatments are often not available, early detection of an eventual infection and its level becomes of outmost importance. Nanomaterials and nanotechnological approaches are increasingly used in the field of viral sensing to address issues related to signal-to-noise ratio, limiting the sensitivity of the sensor. Superparamagnetic nanoparticles (MPs) present one of the most exciting prospects for magnetic bead-based viral aggregation assays and their integration into different biosensing strategies as they can be easily separated from a complex matrix containing the virus through the application of an external magnetic field. Despite the enormous potential of MPs as capture/pre-concentrating elements, they are not ideal with regard of being active elements in sensing applications as they are not the sensor element itself. Even though engineering of magneto-plasmonic nanostructures as promising hybrid materials directly applicable for sensing due to their plasmonic properties are often used in sensing, to our surprise, the literature of magneto-plasmonic nanostructures for viral sensing is limited to some examples. Considering the wide interest this topic is evoking at present, the different approaches will be discussed in more detail and put into wider perspectives for sensing of viral disease markers.

## 1. Introduction

**Figure 1:**
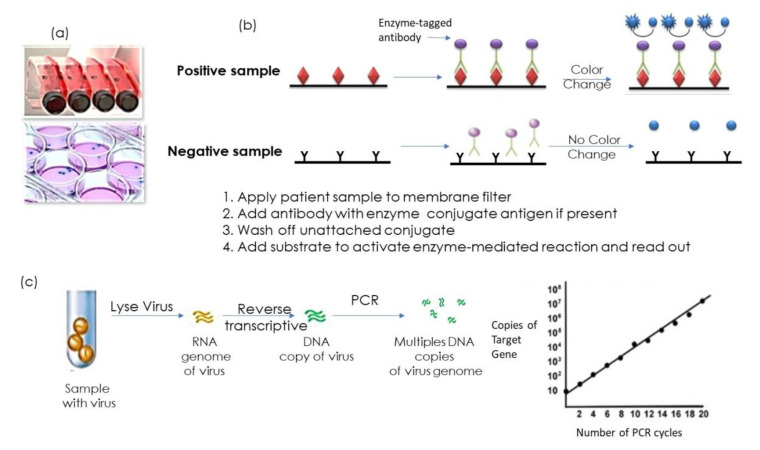
Viral analysis methods: (**a**) Viral growth in vitro in flat (horizontal) flasks or Petri dish; (**b**) enzyme-linked immunosorbent assay (ELISA)-based testing for viral antigens based on the use of a few drops of diluted patient sample applied to a membrane filter previously modified with viral specific antibodies and internal controls. Enzyme-linked antibody conjugate is added to the filter, with the targeted antibody attached to the antigen (in the case of a positive test). Excess conjugate is washed off the filter. Substrate is added to activate the enzyme-mediated reaction to reveal the color change of a positive test. (**c**) Reverse transcription polymerase chain reaction (PCR) for the detection of RNA virus together with the PCR diagram showing the correlation between the number of PCR cycles and the number of copies of target gene detected.

Infectious diseases pose an omnipresent threat to global and public health, as currently evidenced with the newly emerged Coronavirus 2019 (COVID-19) outbreak, associated with several respiratory distress. Generally speaking, the detection of the presence of viral particles is based on the use of a variety of methods commonly operated by specialized laboratories. These include protocols for cell culture with time spans of 2–10 days depending on the virus, immune assay procedures such as enzyme immunoassays (EIA), enzyme-linked immunosorbent assay (ELISA), and fluorescent antibody tests (FAT) [1,2,3,4] (Figure 1). Taking the example of herpes simplex virus (HSV), a double stranded DNA virus from the herpesviridae family, one of the traditional ways for HSV diagnosis involves the monitoring of cytopathic effects of the virus on cultured cells with results taking up to one week to be completed (Figure 1a) [5]. To gain time, enzyme-linked immunosorbent assay (ELISA)-based diagnostic procedures, requiring typically several hours are preferentially used. These assays remain, however, not only labor intensive, but suffer from poor sensitivity (Figure 1b). Due to the need of specific equipment and trained personal, it might require patient samples to be transported to centralized diagnostic laboratories for testing. These facts increase time to answer and costs, while reducing the quality of patient care.

This also applies to the gold standard in clinical setting for the detection of emerging pathogens, polymerase chain reaction (PCR) and reverse transcription PCR (RT-PCR) (Figure 1c). The typical turnaround time for PCR remains >6 h and the approach is based on viral nucleic acid extraction from samples and their transcription into cDNA, which is then amplified and detected using fluorescence or luminescence [6]. In case of viruses having RNA (ribonucleic acid) as genetic material (e.g., SARS-CoV-1 (Severe acute respiratory syndrome coronavirus), SARS-CoV-2, MERS-CoV (Middle East respiratory syndrome–related coronavirus), hepatitis C, Ebola, Dengue virus, etc.), cDNA is first produced from the RNA sample by reverse transcription. RT-PCR is consequently the currently used method for the screening for SARS-CoV-2 infection in nasopharyngeal aspirates/throat and nose swabs in a hospital setting.

The employment of PCR techniques for virus detection and quantification offers the advantages of high sensitivity and reproducibility, combined with an extremely broad dynamic range. While PCR is generally more sensitive than cell culturing and ELISA, it is rather expensive (e.g., equipment ranging from 15,000–90,000 euros depending on equipment sensitivity and through-put capacity with about 30 Euros per assay per patient). In the case of influenza A virus, RT-PCR achieved a 10^3^ times higher sensitivity than virus isolation and 10^6^ to 10^7^ times than ELISA. The detection rate in clinical samples was 93% for RT-PCR, clearly larger than that attained by virus isolation (80%) and ELISA (62%) [7].

**Figure 2:**
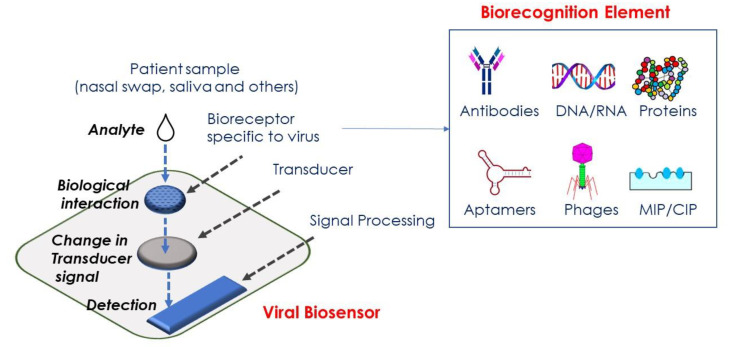
Viral biosensor concept based on using bioreceptors specific to virus (antibodies, RNA, DNA, proteins, aptamers, eventually phages and molecular/chemically imprinted polymers (MIP/CIP)) immobilized on the transducer (optical, electrochemical, etc.) surface. Addition of a viral sample results in biological interaction with the viral bioreceptor on the transducer surface, leading to a change in signal which is processed and results in qualitative and quantitative data.

To improve therapy uptake, new assays are needed to enable rapid diagnosis in a point-of-care (POC) setting. Biosensors are ideal point-of care devices for the diagnosis of viral infections. A biosensor is a device that is based on the immobilization of a biological recognition element onto a solid surface, the transducer, which upon viral binding emits a characteristic signal change (Figure 2) [8,9].

This measurable signal, which can be optical, electrical, electrochemical etc. is used for qualitative and quantitative determination of the viral load. The biological recognition elements for virus sensors range from viral antibodies [10,11,12], viral aptamers [13], to complementary DNA receptors [14], and molecular imprinted polymers [15,16]. Table 1 lists a range of different biosensor platforms for viral detection. Bioanalytical testing often requires a pretreatment stage involving filtration and/or purification [17]. In this context, superparamagnetic particles (MPs) provide a rapid way to pre-concentrate and separate viral particles from complex media. Before more in-depth discussion of the magneto-optical nanostructures, some words about the utilization of MPs in viral sensing are necessary.

## 2. Magnetic Nanoparticles for Viral Sensing

Several types of iron oxide have been used for the synthesis of superparamagnetic nanoparticles with Fe_3_O_4_ (magnetite) and γ-Fe_2_O_3_ (maghemite) being the most popular candidates. They are readily available through a number of synthetic strategies [33,34] and commercial sources, offering a range of different surface functions suitable for separation. Uncoated MPs have limited use. The large surface to volume ratio and, therefore, high surface energy favors particles’ aggregation to minimize their surface energy. Most importantly, magnetite nanoparticles have high chemical activity and oxidize readily when exposed to air and aqueous media with a loss of magnetic properties and dispersibility. To address such issues, the development of a proper surface coating to protect and maintain the stability of magnetic iron oxide NPs is required [17,35,36,37,38]. The success in using these types of nanostructures for viral sensing is likewise related to proper surface functionalization and attachment of viral biorecognition elements such as virus-specific peptides [17], or antibodies [39,40]. The linkage for these ligands is based on the use of classical ligand attachment procedures: use of streptavidin modified beads, which can interact with biotinylated probes; covalent linkage using the Michael addition between thiolated antibodies and vinyl-sulfonate modified beads [39]; covalent integration via protein G coupling attached to dextran coated magnetic beads through 3-(2-pyridyldithio)propionic acid N-hydroxysuccinimide ester linkage [40].

**Figure 3:**
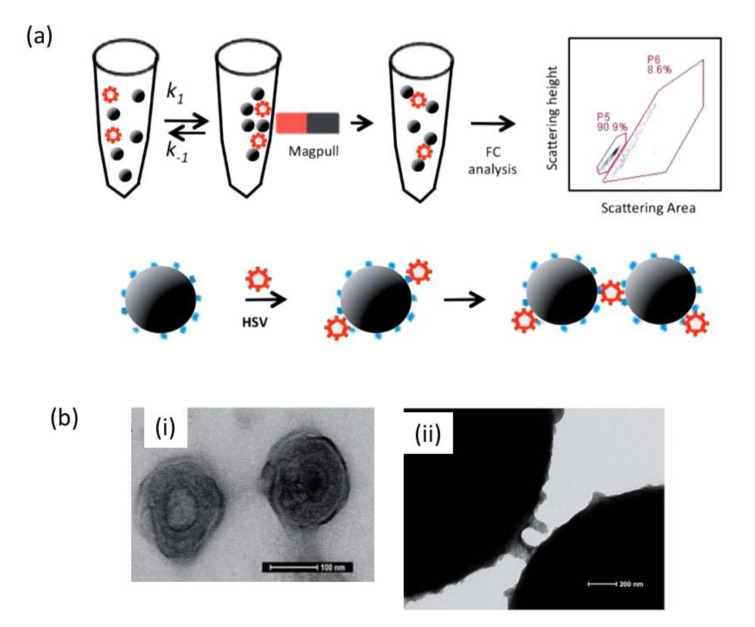
Magnetic particle aggregation assay: (**a**) Workflow of the magnetic particle aggregation assay for HSV-1 detection based on single particle light scattering: anti-HSV-1 modified superparamagnetic nanoparticles (MPs) were incubated with the virus and the aggregation of the beads was achieved by application of a magnetic field. The final aggregation state of the beads was determined using flow cytometry. (**b**) (i) Transmission electron microscopy image of herpes simplex viruses stained with uranyl acetate showing the three characteristic layers of these large viruses and (ii) transmission electron microscopy (TEM) images of magnetic particles modified with biotinylated synthetic peptide after incubation with HSV-1 (reprint from [17], with permission from RSC, 2014).

The work of Lee and his team is one excellent demonstration of the interest of using streptavidin-modified magnetic beads modified with a biotinylated synthetic peptide for the sensing of HSV-1 in less than 30 min with a sensitivity as low as 200 viral particles/mL using flow cytometry (Figure 3a) [17].

The same team reported on the rapid detection of dengue virus in serum using magnetic separation and fluorescence detection [39]. The assay was based on the use of MPs and fluorescent microparticles modified with an anti-type 2 dengue virus monoclonal antibody. Separation of the MPs from the serum, based on their magnetophoretic mobility, allowed detection of the dengue virus with a LoD of 10 pfu/mL within 15 min. Monodisperse magnetic nanoparticles conjugated with virus-surface-specific antibodies have also been shown to form supramolecular architectures with enhanced magnetic properties, as detected by magnetic resonance methods (nuclear magnetic resonance (NMR)/magnetic resonance imaging (MRI)) [40]. The observed magnetic relaxation changes that occur upon viral-induced assembly allowed for highly sensitive and selective detection of a virus in complex biological media, as exemplified by the detection of adenovirus-5 and herpes simplex virus-1 at concentrations of 50 viral particles/100 μL without the need of extensive sample preparation. MPs were also very recently proposed to combine virus lysis and binding steps into one step, for subsequent RT-PCR analysis of COVID-19 samples [41]. In this example, the MPs allowed purification of viral RNA from multiple samples within 20 min. Magnetic nanoparticles-based detection tests are currently explored for the extraction of RNA from a solution containing a patient sample [42]. These particles, upon coating with a silica thin layer, exhibit a strong affinity for RNA. The solution contains substances that open the virus envelope so that its genetic material can be released and extracted. RNA from the virus in the solution is strongly attracted to the silica-covered magnetic nanoparticles. The next step was to use a magnet to pull the RNA-covered particles out of the solution and subsequently identify the genetic code from the RNA and compare it to the coronavirus.

## 3. Magneto-Plasmonic Nanoparticles for Sensing

Despite the enormous potential of MPs as capture/preconcentrating elements in viral sensing systems, these materials are not ideal with regards to being active elements in sensing applications and are not the sensor element itself [37]. The engineering and design of nanoparticles with multifunctional characteristics are important for making magnetic nanoparticles more directly applicable for biosensors (i.e., the core of the sensing system) and for viral sensing. Magnetic-plasmonic nanoparticles are in this respect regarded as emerging and highly promising materials for advanced biosensing if their strong magnetic response is preserved along with a sharp plasmonic signal. The combination of the magnetic properties of MPs with the plasmonic signature of gold nanoparticles (Au NPs) is particularly attractive for analytical science as the strong extinction band in the ultraviolet (UV)–visible region of Au NP allows Au NPs@MPs to be used as localized surface plasmon resonance (LSPR sensors), while the resonance light scattering properties make them ideal platforms for surface-enhanced Raman spectroscopy (SERS) sensing [43,44]. The physical origin of the strong light absorption by Au NPs is due to the coherent oscillation of the conduction electrons induced by the interacting electromagnetic field. The plasmon frequency is highly sensitive not only to the size, shape and composition of the Au NPs, but also to the dielectric medium, allowing ligand–analyte interactions to be followed visually due to colorimetric changes.

Various strategies have been designed to fabricate diverse magneto-plasmonic nanostructures by growing Au shells over the MPs or by attaching isolated Au nanoparticles (Au NPs) onto the surface of MPs [37,45,46,47,48,49,50]. The obtained nanocomposites exhibit the typical core–shell “gold-coated magnetic” nanostructures. The saturation magnetization of such nanostructures dramatically decreases with the increase in the plasmonic component, because of the inherent magnetic shielding effect of Au shell deposited on the surface of the MPs. Although this limitation can be alleviated partially by reducing the thickness of the plasmonic component, the corresponding plasmonic response is in this case largely compromised. So where do we stand when it comes to viral sensing with these hetero-nanostructures (Table 2)?

**Figure 4:**
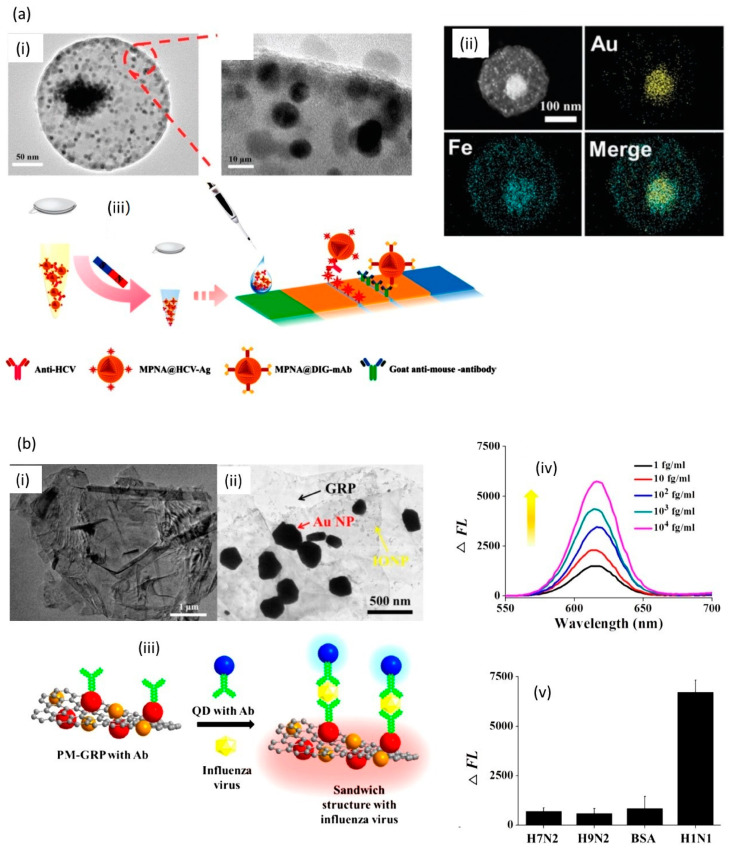
(**a**) (i) TEM image of a magneto-plasmonic particle, (ii) high-angle annular dark-field scanning transmission electron microscopy (HAADF-TEM) and energy-dispersive X-ray spectrometry (EDS) elemental mapping, (iii) HCV detection scheme based on anti-HCV modified MPs, followed by magnetic separation from the medium, addition to LFIA strip platform sensing using a double antigen sandwich lateral flow immunoassay (LFIA) strip platform (reprint from [51], with permission from Wiley, 2019 (**b**) TEM images of (i) graphene, (ii) graphene modified with gold nanoparticles (Au NPs) and MPs, (iii) Influenza virus detection scheme using antibody modified fluorescent quantum dots (QDs) for read out, (iv) Change of fluorescence intensity depending on virus concentration, (v) selectivity test against other influenza virus types (reprint from [11], with permission from Elsevier, 2018).

Hao et al. fabricated magnetic-plasmonic nano-assemblies with highly retained magnetic-plasmonic activities [51]. The concept was based on co-assembling oleic acid-coated MPs (OC-MPs) with oleylamine-coated Au NPs (OA-Au NPs) to form colloidal magnetic-plasmonic nano-assemblies exhibiting a typical core-shell heterostructure, comprising aggregated OA-Au NPs as a plasmonic core surrounded by an assembled magnetic shell of OC-MPs (Figure 4a). Owing to the high loading of OA-Au NPs and reasonable spatial distribution of OC-MPs, the resultant assembly exhibited highly retained magnetic–plasmonic activities simultaneously. The large-area transmission electron microscopy (TEM) images in Figure 4a show the spherical nature of the particles with characteristic core-shell structures of 225 nm average size. The high-angle annular dark-field scanning transmission electron microscopy (HAADF-STEM) and energy-dispersive X-ray spectrometry (EDS) elemental mapping underline that Au is centered in the core, whereas Fe elements are uniformly and homogenously distributed in the outer shell of the magneto-plasmonic nanostructures. The feasibility of the probe was assessed for the sensing of HCV antibodies using a lateral flow immunoassay (LFIA) test strip. It consists on the conjugation of carboxylated magneto-plasmonic nanostructures with hepatitis C virus antigen (HCV-antigen) via 1-ethyl-3-(3-dimethylaminopropyl)carbodiimide (EDC)-mediated covalent coupling. The detection principle (Figure 4a) relies on capturing anti-HCV from serum samples on the particles, magnetic collection, and resuspending in phosphate-buffered saline (PBS) to run the LFIA test strip that was prepared by dispersing HCV antigen and goat-anti-mouse immunoglobuline G. The production of a red band at the T line was used for anti-HCV quantification [51]. This approach resulted in a detection limit of 0.24 pg mL^−1^ for anti-HCV.

The concept of tuning the fluorescence properties of a quantum dot in the presence of plasmonic particles for viral detection is well-known and was used for the detection of Zika virus using plasmonic silver/gold nanoparticles to mediate the fluorescence signal of glutathione-capped CdSeS nanocrystals in a molecular beacon biosensor [52]. In this case, the extent of the fluorescence enhancement based on Zika RNA detection is proportional to the LSPR-mediated fluorescence signal with a limit of detection of 1.7 copies/mL. A comparable approach has allowed researchers to detect and discriminate between the various serotypes of dengue virus (DENV) using (GSH)-functionalized CdSe/ZnSeS core/alloyed shell quantum dots conjugated to Au NPs with detection limits of the serotypes ranging from 31–260 copies per mL [53].

A LSPR-amplified magneto fluoro-immunoassay was used by the group of Park for the detection of one of the major causes of the infectious and contagious gastroenteritis, the norovirus (NoV) [46]. The assay is based on the utilization of magneto-plasmonic hybrid particles and fluorescent CdSeS quantum dots (QDs), both modified with anti-norovirus genomgroup II antibody for virus separation, first, followed by detection via LSPR-based plasmonic resonance energy transfer. Anti-NoV antibodies were immobilized onto the magneto-plasmonic hybrid particles by, first, modification of the gold shell by 11-mercaptaundecanoic acid followed by EDC/NHS (1-ethyl-3-[3-dimethylaminopropyl]-carbodiimide hydrochloride/ N-Hydroxysuccinimide) coupling of the antibody. In the absence of the virus, no fluorescence signal was recorded under light excitation of the particle’s mixture. The intensity of the light emission in presence of virus scales with its concentration. The target norovirus-like particles can be detected in the range of 1 pg/mL to 5 ng/mL with a limit of detection of 0.48 pg/mL in feces.

The same group demonstrated that graphene modified with Au NPs as well as MP NPs results in a sensitive magneto-plasmonic hybrid for the magnetic fluoro-immunosensing (MFIS) of influenza virus [11] (Figure 4b). This assay was based on the use of anti-hemagglutinin antibody modified Au NPs which were further functionalized with fluorescence quantum dots. The antibodies were integrated onto the Au NPs by linkage of cysteamine via its thiol groups followed by EDC/NHS covalent coupling to the antibody. In the presence of influenza virus, a sandwich structure between the quantum dots and the plasmon-magnetic graphene was formed with the fluorescence of the QDs scaling linearly with the virus concentration. Influenza virus A (H1N1) in deionized water was successfully detected with a detection limit of 7.27 fg/mL.

Due to the good conductivity of gold, Au-coated MPs provide a practical platform for electrochemical assays [45,47,59,60]. While the concept was not investigated for viral sensing, it could be applied easily by using the concept developed by Gooding et al. called “dispersible electrodes” [45]. This concept relies on the dispersion of ligand-modified Au@MNPs into the solution containing the analyte, where it is captured by selective binding. Thereafter, a magnetic field is applied to direct the nanoparticles to a conducting surface to detect the analyte. This concept was first tested for Cu^2+^ sensing [45] and later for the detection of prostate-specific antigen (PSA) [47] with a detection limit of 100 fg mL^−1^.

**Figure 5:**
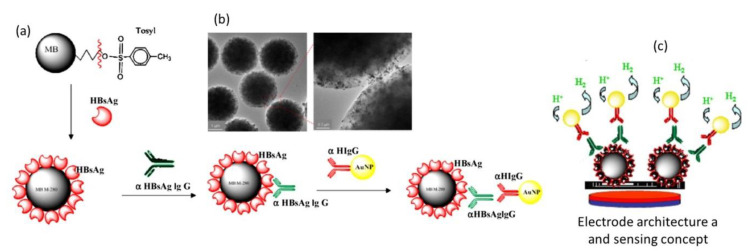
Electrochemical magneto-immunosensor for the sensing of hepatitis B IgG antibodies: (**a**) schematic of the assay: MPs were modified with hepatitis B antigens (HBsAg), Au NPs were modified with polyclonal anti-human IgG (α-HIgG), (**b**) TEM image of the MPs/HBsAg/α-HBsAg IgG/α-HIgG/Au NPs complex formed and the interface between a MP and Au NPs (small black points), (**c**) sensing concept based on the dosing of the amount of Au stripped from the assembly in 1 M HCl at −1 V for 5 min. (reprint from [12], with permission from Elsevier, 2010.)

Electrochemical impedance (EIS)-based sensing of hepatitis B virus (HBV) was achieved using a carbon paste electrode modified MPs and Au NPs in the presence of HBV specific DNA probe [60]. The proposed DNA biosensor could measure target HBV DNA virus concentration down to 0.31 pM, through a change in the interfacial charge transfer resistance (R_CT_). An Au NPs-based electrochemical magneto-immunosensor was developed by the team of Merkoci for the detection of anti-hepatitis B virus antibodies in human serum (Figure 5) [12]. In this system, tosyl-activated MPs of 2.8 µm in size (Figure 5a) conjugated to hepatitis B antigens allowed capturing hepatitis B IgG antibodies. The addition of Au NPs modified with polyclonal anti-human IgG results in a second immunoreaction and gives rise to the formation of a large complex where the quantity of Au NPs is proportional to the concentration of the hepatitis B IgG antibodies. The electrochemical detection of Au NPs was carried out by exploiting their catalytic properties towards the hydrogen evolution reaction (HER) in an acidic medium, without nanoparticles’ dissolution.

## 4. Perspectives and Conclusions

The application of magneto-plasmonic nanoparticles for biosensing has seen some successful examples including viral sensing. These hybrid nanomaterials are an outcome of the progress made in the synthesis and characterization of these materials as well as proper ligand attachment. The possibility to apply diverse surface chemistry approaches to provide functional groups and surface ligands onto these hybrids makes these nanostructures directly applicable for viral sensing. The combination of pre-concentration, separation and capture of the analyte together with sensing allows the lowering the detection limit significantly. Fast and sensitive screening will permit early therapy and monitoring of positively diagnosed patients, thus saving suffering and lives. While the direct benefit of such diagnostic devices is evident, there is still a considerable amount of work to be performed in terms of optimizing viral biosensors and magneto-plasmonic viral detection strategies. The lack of clinical validation of most of the proposed sensing concepts is a large limitation, as only when these sensors enter into clinical testing, their real performance can be validated and eventually counter measure will be taken to improve their sensitivity and selectivity. Viral biosensors and magneto-plasmonic viral sensing concepts will not substitute PCR, but can be seen as an important additional fast approach to decide upon the presence of the virus in a patient and whether he needs to be isolated, treated, etc. The main hurdle to overcome in this field is linked to industrial upscaling and standardization. Until then, these different sensing concepts will remain in research laboratories and might not be taken further. Having said this, magneto-plasmonic particles still have large scientific interest as the optical addressability of these particles will allow for SERS-based viral sensing. While no SERS-based viral sensors are available in the literature, Hong et al. used Fe_3_O_4_@Au nanostructures for bacterial cells capture and label-free SERS sensing of bacterial cell molecular structures based on optical fingerprints [55]. Dual-recognition SERS sensors for bacteria detection using vancomycin SERS tags and aptamer Fe_3_O_4_@Au NPs were reported by Pang et al. [57]. The SERS active aptamer Fe_3_O_4_@Au NPs allowed bacteria enrichment, while the vancomycin-modified Au NPs permitted pathogen sensing down to 3 cells/mL with a wide dynamic range to 10^7^ cells/mL in 50 min. Another interesting concept is to take advantage of the enzyme-mimicking activity of iron oxide-based nanostructures. It is only through the combination of efforts from materials scientists, virologists, clinicians, governmental bodies and anybody interested in the field of viral sensing that will advance this exciting and highly important field further.

## Figures and Tables

**Table 1:** Selected examples of biosensor platforms for viral detection.

Sensor Type	Virus	Surface Ligand	LoD ^1^	Linear Range	Ref.
Electrochemical	HIV-1	MIP of HIV aptamer	0.3 fM	3 fM–0.3 nM	[15]
Electrochemical	HIV, HBV	streptavidin	50 pM	2.53–50.60 nM/mL	[18]
Electrochemical	Ebola	biotinylated-DNA	4.7 nM	-	[19]
Electrochemical	Zika	envelope protein antibody	<10 pM	10 pM–1 nM	[20]
Electrochemical	Norovirus	DNA	8.8 pM	1 pM–10 nM	[21]
Electrochemical	Influenza A	DNA	0.5 nM	1–10 nM	[22]
Electrochemical	Dengue	DNA	2.7 pM	10 pM–10 µM	[23]
Electrochemical	HPV 16E7	RNA aptamer	0.1 ng/mL	0.2–2 ng/mL	[13]
Electrochemical	HRV	MIP	-	3 µg/mL–3 g/mL	[16]
Electrical	SARS-CoV-2	antibody	2.42 × 10^2^ copies/mL	10^2^–10^4^ copies/mL	[10]
Optical	HIV-1	Glycoprotein-120 antibody	10^5^ copies/mL	10^4^–10^8^ copies/mL	[24]
Optical	HVC	DNA stand	0.1 fM	0.1 fM–6 µM	[25]
Optical	Influenza A	anti-HA	5 pg/mL	5 ag/mL–5 µg/mL	[26]
Optical	Ebola	DNA	0.2 pfu/mL	0.21–1.05 × 10^−5^ pfu/mL	[27]
Optical	Zika	envelope protein	5 pfu/mL	5–500 pfu/mL	[28]
Optical	Norovirus	anti-norovirus antibody	0.01 ng/mL	0.01–100 ng/mL	[29]
Optical	Influenza H5N1	aptamer	3.5 ng/mL	2–200 ng/mL	[30]
Optical	Dengue	antigen	/	/	[31]
Optical	HPV 16E7	anti-HPV 16E7	2.87 ng/mL	0.021–15 ng/mL	[32]

^1^ LoD: limit of detection. Anti-HA: anti-hemagglutinin. HVC: hepatitis virus C. HIV-1: human immunodeficiency virus. HRV: human rhinovirus. HPV: human papilloma virus. MIP: molecularly imprinted polymer. SARS: severe acute respiratory syndrome.

**Table 2:** Magneto-plasmonic based strategies for viral and bacterial sensing.

Sensor Type	Analyte	Particle Type	Limit of Detection	Linear Range	Ref.
Optical (fluorescence)	Norovirus	Au/Fe_3_O_4_ and CdSeS QDs	0.48 pg/mL	1 pg/mL–5 ng/mL	[46]
Optical	HCV	Au/Fe_3_O_4_	0.24 pg/mL	0.24–120 pg/mL	[51]
Electrochemistry	HBV	Fe_3_O_4_ and Au NPs	-	-	[12]
Optical (fluorescence)	Influenza H1N1	Au/Fe_3_O_4_ decorated graphene	7.27 fg/mL	10–10^4^ fg/mL	[11]
Electrochemistry	HBV	Fe_3_O_4_ and Au NPs	83 pM	8 3 pM–64 µM	[54]
SERS	*E. Coli*	Au/Fe_3_O_4_	10^5^ cfu/mL	/	[55]
SERS	*S. Aureus*	MnFe_2_O_4_@Au	10 cfu/mL	10^1^–10^5^ cfu/mL	[56]
SERS	*S. Aureus*	Au/Fe_3_O_4_	3 cfu/mL	10–10^7^ cfu/mL	[57]
SPR	Tuberculosis	Au/Fe_3_O_4_	0.1 ng/mL	0.1–100 ng/mL	[58]

HBV: hepatitis B virus. HCV: hepatitis C virus.
